# Supplementary material for: Computational Insight into the Nature and Strength of the π-Hole Type Chalcogen∙∙∙Chalcogen Interactions in the XO2∙∙∙CH3YCH3 Complexes (X = S, Se, Te; Y = O, S, Se, Te)
Source: Int J Mol Sci. 2023 Nov 10;24(22):16193. doi: 10.3390/ijms242216193 (PMC10671658; doi:10.3390/ijms242216193)
Supplement: Supplementary file 1 [file ijms-24-16193-s001.zip › ijms-2692285-supplementary.pdf]

Computational Insight into the Nature and Strength of the π-Hole Type Chalcogen···Chalcogen Interactions in the $XO_2\cdots CH_3YCH_3$ Complexes (X = S, Se, Te; Y = O, S, Se, Te)

Fengying Lei [1], Qingyu Liu [1], Yeshuang Zhong [1], Xinai Cui [1], Jie Yu [1], Zuquan Hu [1], Gang Feng [2], Zhu Zeng [1,*] and Tao Lu [1,*]

[1] School of Basic Medical Sciences/School of Biology and Engineering, Guizhou Medical University, Guiyang 550025, PR China
[2] School of Chemistry and Chemical Engineering, Chongqing University, Daxuecheng South Rd. 55, Chongqing 401331, PR China

*** Corresponding authors:**

E-mail address: lutao0409@gmc.edu.cn (T. L.); zengzhu@gmc.edu.cn (Z. Z.)

Contents:

Supplementary figures

Figure S1. Correlation plots between the binding energy (E_B) and the most positive electrostatic potentials ($V_{S,max}$) on the X atom of the XO_2 (X = S, Se, and Te).
Figure S2. Correlation plots between the binding energy (E_B) and the most negative electrostatic potentials ($V_{S,min}$) on the Y atom of the CH_3YCH_3 (Y = O, S, Se, and Te).
Figure S3. Correlation plots between the binding energy (E_B) and the X···Y distance between two interacting chalcogen atoms, X = S, Se, Te; Y = O, S, Se, and Te.
Figure S4. Correlation plots between the binding energy (E_B) and the total interaction energies (E_{total}) of the studied complexes obtained by the SAPT analysis.

Supplementary tables

Table S1. Cartesian coordinates of the $SO_2\cdots CH_3YCH_3$ (Y = O, S, Se, Te) complexes in its principal inertial axis system at the MP2/aug-cc-pVTZ(PP) level of theory.

Table S2. Cartesian coordinates of the $SeO_2\cdots CH_3YCH_3$ (Y = O, S, Se, Te) complexes in its principal inertial axis system at the MP2/aug-cc-pVTZ(PP) level of theory.

Table S3. Cartesian coordinates of the $TeO_2\cdots CH_3YCH_3$ (Y = O, S, Se, Te) complexes in its principal inertial axis system at the MP2/aug-cc-pVTZ(PP) level of theory.

Table S4. The geometrical parameters associated with the C−H···O hydrogen bonds in the twelve studied complexes.

Supplementary figures

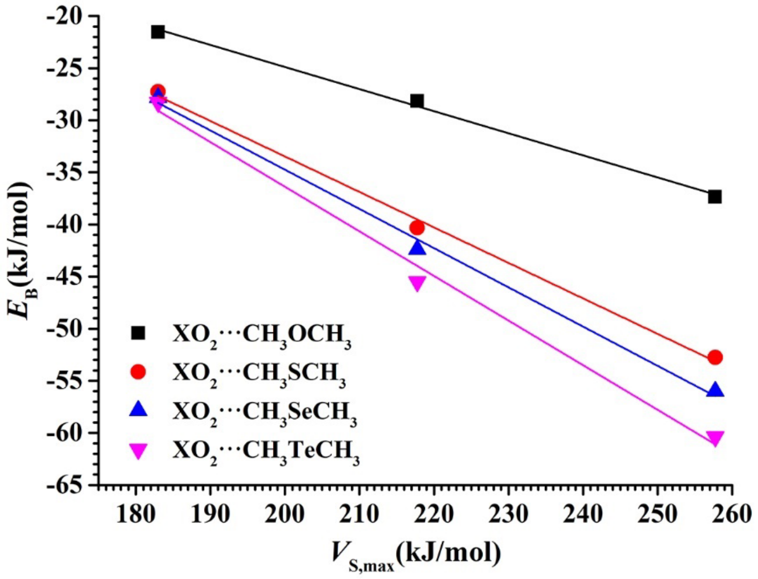

Figure S1. Correlation plots between the binding energy (E_B) and the most positive electrostatic potentials ($V_{S,max}$) on the X atom of the XO_2 (X = S, Se, and Te).

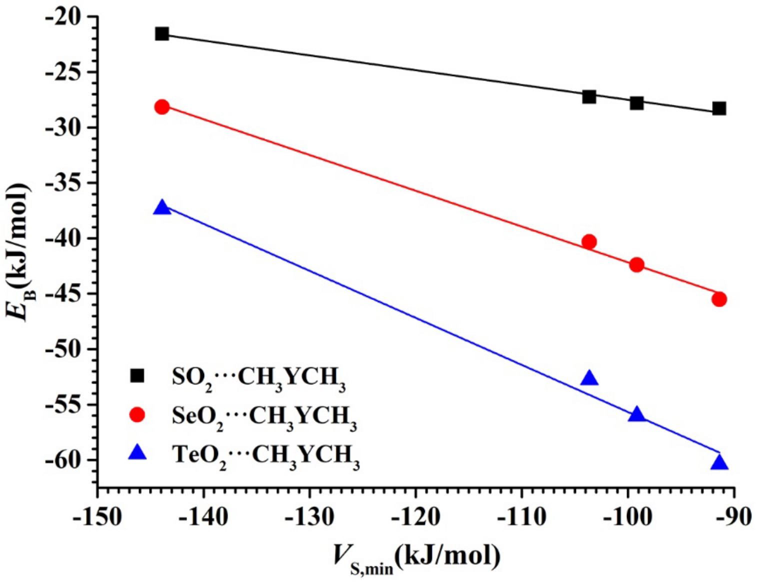

Figure S2. Correlation plots between the binding energy (E_B) and the most negative electrostatic potentials ($V_{S,min}$) on the Y atom of the CH_3YCH_3 (Y = O, S, Se, and Te).

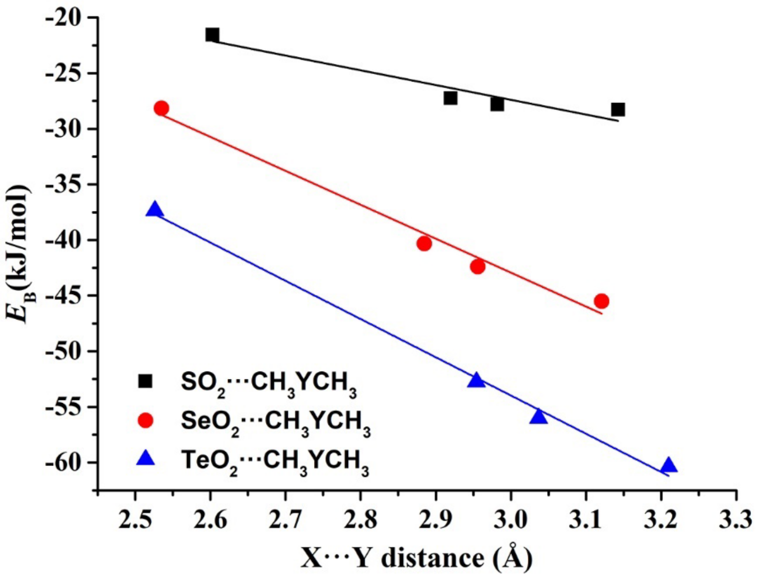

Figure S3. Correlation plots between the binding energy (E_B) and the X···Y distance between two interacting chalcogen atoms, X = S, Se, Te; Y = O, S, Se, and Te.

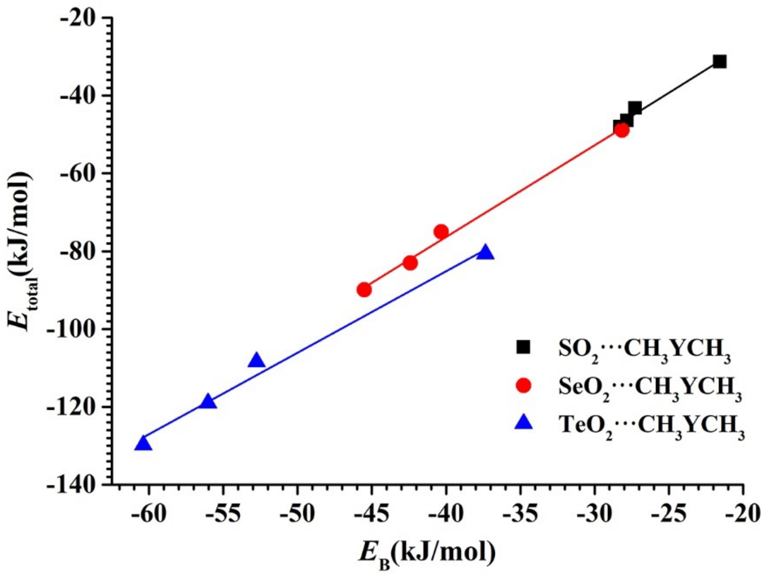

Figure S4. Correlation plots between the binding energy (E_B) and the total interaction energies (E_{total}) of the studied complexes obtained by the SAPT analysis.

Supplementary tables

Table S1. Cartesian coordinates of the $SO_2 \cdots CH_3YCH_3$ (Y = O, S, Se, Te) complexes in its principal inertial axis system at the MP2/aug-cc-pVTZ(PP) level of theory.

$SO_2 \cdots CH_3OCH_3$				$SO_2 \cdots CH_3SeCH_3$			
Atom	*a* (Å)	*b* (Å)	*c* (Å)	Atom	*a* (Å)	*b* (Å)	*c* (Å)
S	-1.270245	-0.246643	-0.338087	S	-1.807245	-0.000079	-0.402139
O	-1.067288	-1.113844	0.827027	O	-1.930383	-1.251497	0.364791
O	-1.599689	1.157858	-0.085741	O	-1.930481	1.251366	0.364725
O	1.282686	0.223707	-0.526221	C	1.107937	-1.440057	0.823153
C	1.948593	-0.984857	-0.186051	H	1.261668	-2.365832	0.275180
H	1.773170	-1.688276	-0.995383	H	1.905155	-1.303259	1.548888
H	3.021839	-0.809746	-0.080314	H	0.130985	-1.458036	1.304295
H	1.552531	-1.394551	0.747172	C	1.107493	1.439687	0.823542
C	1.470289	1.204503	0.486085	H	1.904673	1.302913	1.549330
H	2.531980	1.439040	0.591257	H	1.261045	2.365737	0.275970
H	0.923552	2.091761	0.180944	H	0.130523	1.457382	1.304662
H	1.081885	0.848424	1.444999	Se	1.174006	0.000165	-0.457071

$SO_2 \cdots CH_3SCH_3$				$SO_2 \cdots CH_3TeCH_3$			
Atom	*a* (Å)	*b* (Å)	*c* (Å)	Atom	*a* (Å)	*b* (Å)	*c* (Å)
S	-1.519461	-0.000876	-0.376519	S	-2.072248	0.001722	-0.450824
O	-1.567798	-1.251504	0.396506	O	-2.246667	-1.251965	0.307779
O	-1.568576	1.251454	0.393629	O	-2.242055	1.253900	0.311405
C	1.505319	-1.369937	0.499770	C	0.760321	-1.555364	1.058818
H	1.521828	-2.287681	-0.083207	H	1.078729	-2.484812	0.594294
H	2.426573	-1.284124	1.072331	H	1.368000	-1.351309	1.935868
H	0.638595	-1.382036	1.160926	H	-0.299427	-1.603264	1.305831
C	1.502703	1.370064	0.502152	C	0.766827	1.557263	1.054098
H	2.423624	1.284145	1.075300	H	1.377365	1.356200	1.929843
H	1.519046	2.288766	-0.079280	H	1.083875	2.485049	0.585309
H	0.635602	1.380201	1.162861	H	-0.292059	1.606520	1.304661
S	1.386810	0.000900	-0.663578	Te	1.068969	-0.001208	-0.347569

Table S2. Cartesian coordinates of the $SeO_2\cdots CH_3YCH_3$ (Y = O, S, Se, Te) complexes in its principal inertial axis system at the MP2/aug-cc-pVTZ(PP) level of theory.

$SeO_2\cdots CH_3OCH_3$			
Atom	*a* (Å)	*b* (Å)	*c* (Å)
O	-1.528328	-0.229060	-0.525227
C	-1.777153	-1.217891	0.474410
H	-1.217730	-2.105060	0.194399
H	-1.438070	-0.864598	1.451730
H	-2.844977	-1.438839	0.511336
C	-2.179089	0.996736	-0.196819
H	-1.796320	1.390782	0.747650
H	-1.967533	1.697295	-1.000009
H	-3.256070	0.835991	-0.124386
O	1.273819	-1.375702	0.083362
O	0.712459	1.076418	1.077037
Se	0.958663	0.177591	-0.250813

$SeO_2\cdots CH_3SeCH_3$			
Atom	*a* (Å)	*b* (Å)	*c* (Å)
O	-1.566012	-1.349880	0.610474
O	-1.566153	1.349641	0.611046
C	1.440638	-1.447737	0.812541
H	1.679667	-2.356351	0.266795
H	2.198345	-1.255702	1.567280
H	0.442116	-1.527264	1.247720
C	1.440865	1.448065	0.811922
H	2.198957	1.256602	1.566418
H	1.679526	2.356468	0.265643
H	0.442493	1.527647	1.247437
Se	1.463200	-0.000118	-0.456918
Se	-1.488871	0.000075	-0.298383

$SeO_2\cdots CH_3SCH_3$			
Atom	*a* (Å)	*b* (Å)	*c* (Å)
O	-1.189134	-1.349388	0.629701
O	-1.191472	1.349089	0.628893
C	1.820542	-1.376398	0.487951
H	1.897234	-2.283226	-0.106955
H	2.723457	-1.246934	1.080463
H	0.933106	-1.436546	1.121560
C	1.818600	1.376877	0.488731
H	2.721985	1.248786	1.080868
H	1.893544	2.284436	-0.105311
H	0.931507	1.435189	1.123020
S	1.666612	0.000811	-0.661314
Se	-1.192842	-0.000446	-0.280631

$SeO_2\cdots CH_3TeCH_3$			
Atom	*a* (Å)	*b* (Å)	*c* (Å)
O	-1.888219	-1.352177	0.575435
O	-1.886867	1.352363	0.576351
C	1.103009	-1.561326	1.051973
H	1.485914	-2.472820	0.601296
H	1.675142	-1.313171	1.941599
H	0.034944	-1.656504	1.265015
C	1.104802	1.561938	1.050674
H	1.677964	1.314665	1.939874
H	1.486970	2.473047	0.598607
H	0.036953	1.657154	1.264856
Te	1.356631	-0.000360	-0.350369
Se	-1.764379	0.000329	-0.330066

Table S3. Cartesian coordinates of the $TeO_2\cdots CH_3YCH_3$ (Y = O, S, Se, Te) complexes in its principal inertial axis system at the MP2/aug-cc-pVTZ(PP) level of theory.

$TeO_2\cdots CH_3OCH_3$				$TeO_2\cdots CH_3SeCH_3$			
Atom	*a* (Å)	*b* (Å)	*c* (Å)	Atom	*a* (Å)	*b* (Å)	*c* (Å)
O	1.046928	1.597510	0.213041	O	-1.283903	-1.479685	0.811078
O	0.440475	-1.109449	1.284345	O	-1.283952	1.479730	0.810984
O	-1.678355	0.227155	-0.515939	C	1.734449	-1.457556	0.793988
C	-2.022113	1.225540	0.457527	H	2.018929	-2.348565	0.241032
H	-1.404222	2.094739	0.253682	H	2.479765	-1.232938	1.551969
H	-3.079526	1.467487	0.354083	H	0.733631	-1.582480	1.219831
H	-1.813169	0.855050	1.463021	C	1.734435	1.457516	0.794079
C	-2.354418	-1.005996	-0.241373	H	2.479920	1.232973	1.551915
H	-3.430940	-0.835428	-0.251227	H	2.018676	2.348606	0.241127
H	-2.085585	-1.700493	-1.032888	H	0.733669	1.582259	1.220096
H	-2.037055	-1.401921	0.725140	Se	1.706653	0.000019	-0.466604
Te	0.800717	-0.144585	-0.205006	Te	-1.322332	-0.000012	-0.243583

$TeO_2\cdots CH_3SCH_3$				$TeO_2\cdots CH_3TeCH_3$			
Atom	*a* (Å)	*b* (Å)	*c* (Å)	Atom	*a* (Å)	*b* (Å)	*c* (Å)
O	-0.907896	-1.478334	0.818276	O	-1.606008	-1.482467	0.789439
O	-0.908089	1.478359	0.818127	O	-1.605957	1.482441	0.789520
C	2.090255	-1.385378	0.463517	C	1.414055	-1.569509	1.041639
H	2.208002	-2.278814	-0.144807	H	1.825965	-2.466023	0.586403
H	2.986563	-1.221549	1.057202	H	1.985574	-1.297428	1.924712
H	1.199598	-1.489777	1.090146	H	0.347389	-1.697181	1.261349
C	2.089987	1.385470	0.463611	C	1.414147	1.569577	1.041525
H	2.986942	1.222302	1.056501	H	1.985964	1.297716	1.924472
H	2.206457	2.279110	-0.144662	H	1.825755	2.466119	0.586068
H	1.199797	1.489088	1.091014	H	0.347517	1.697078	1.261500
S	1.890149	0.000066	-0.671531	Te	1.608341	-0.000024	-0.361657
Te	-1.030448	-0.000042	-0.229132	Te	-1.600489	0.000015	-0.266712

Table S4. The geometrical parameters associated with the C−H···O hydrogen bonds in the twelve studied complexes.

Complexes	$d_{O\cdots H}$ (Å)	∠C−H···O (°)
$SO_2\cdots CH_3OCH_3$	2.704/2.636	102.4/110.2
$SO_2\cdots CH_3SCH_3$	**2.338** [a]	**123.4**
$SO_2\cdots CH_3SeCH_3$	**2.275**	**129.0**
$SO_2\cdots CH_3TeCH_3$	**2.217**	**138.2**
$SeO_2\cdots CH_3OCH_3$	2.598/2.550	106.0/114.3
$SeO_2\cdots CH_3SCH_3$	**2.181**	**131.1**
$SeO_2\cdots CH_3SeCH_3$	**2.115**	**137.9**
$SeO_2\cdots CH_3TeCH_3$	**2.066**	**146.2**
$TeO_2\cdots CH_3OCH_3$	2.501/2.555	113.7/115.8
$TeO_2\cdots CH_3SCH_3$	**2.126**	**137.1**
$TeO_2\cdots CH_3SeCH_3$	**2.061**	**144.3**
$TeO_2\cdots CH_3TeCH_3$	**2.021**	**151.8**

[a] Both the bond length and bond angle of two C−H···O hydrogen bonds in these complexes are identical due to they have the C_s symmetry.
